# Monitoring Antibiotic Usage in German Dairy and Beef Cattle Farms—A Longitudinal Analysis

**DOI:** 10.3389/fvets.2019.00244

**Published:** 2019-07-26

**Authors:** Katharina Hommerich, Inga Ruddat, Maria Hartmann, Nicole Werner, Annemarie Käsbohrer, Lothar Kreienbrock

**Affiliations:** ^1^Department of Biometry, Epidemiology, and Information Processing, WHO Collaborating Centre for Research and Training for Health at the Human-Animal-Environment Interface, University of Veterinary Medicine, Hanover, Germany; ^2^Department Biological Safety, Federal Institute for Risk Assessment, Berlin, Germany; ^3^Department for Farm Animals and Veterinary Public Health, Institute of Food Safety, Food Technology and Veterinary Public Health, University of Veterinary Medicine Vienna, Vienna, Austria

**Keywords:** monitoring of antimicrobial consumption, treatment frequency, regression modeling, dairy cows, dairy calves, beef cattle

## Abstract

It is well-established that antimicrobial use is a major factor for the development of antimicrobial resistance. To analyze the associations between antimicrobial resistance and usage of antimicrobial agents, data from monitoring and surveillance systems are crucial. Within the project VetCAb (Veterinary Consumption of Antibiotics), antibiotic usage data in German livestock is regularly collected and evaluated. Based on a cross-sectional study in 2011, the project was continued as the longitudinal study VetCAb-Sentinel with ongoing participant recruitment and data collection from 2013. The data collection is based on official German application and delivery forms (ADF), voluntarily provided by veterinarians and farmers. In this study the results of antibiotic usage data of dairy cows, dairy calves and beef cattle were described, using a semi-annual treatment frequency, and 95,944 ADF issued between 2011 and 2015 were analyzed. Results show that the median of the treatment frequency in dairy calf and beef cattle holdings slightly decreased from 0.4 to 0.3 and from 0.2 to 0 days, respectively, whereas the median in dairy cow holdings ranged between 1.9 and 2.3 during the observed period. Temporal changes and the effect of the factors “farm size” and “region” on the treatment frequency were investigated, using multiple linear mixed and logistic regression models. Generally, the factor “time” has a statistically significant impact on the treatment frequency in all production types. In addition, a temporal trend test over the first six half-years shows that an increasing linear trend can be stated in dairy cows and dairy calves (*p* = 0.017; *p* = 0.004, respectively). If the time-period is extended to all eight half-years under study, this turns into a quadratic effect (dairy cows: *p* = 0.006; dairy calves: *p* < 0.001). In dairy calves and beef cattle the factor “farm size” also has a statistically significant impact. The factor “region,” in contrast, shows no statistically significant impact at all. Compared to other livestock populations in Germany, the use of antimicrobials in dairy cows, dairy calves, and beef cattle appears to be low, but varies across several associated factors. Considering these effects, it is recommended that the size of dairy calf and beef cattle holdings is regularly considered in the evaluation of antimicrobial usage data over time.

## Introduction

The impact of the use of antibiotics on antimicrobial resistance (AMR) in food-producing animals has been the subject of increasing public, scientific and political debate in recent years. It is well-known that the development of resistance is related to some extent to the antibiotic use (1, 2). Therefore, for regular evaluation of these associations and for interpretation of resistance patterns and trends, among others, detailed information about antibiotic consumption is needed (3, 4). At the EU level, Directive 2003/99/EC requires the member states to carry out a monitoring of AMR in zoonotic agents and commensal bacteria (5). In Germany, since 2011, the amount of veterinary medicinal products containing antimicrobials delivered to veterinarians by pharmaceutical companies and wholesalers is documented in a central information system and evaluated annually by the Federal Office of Consumer Protection and Food Safety (BVL) (6). Results show that the amount of antibiotics have been reduced by more than half by 2015 (7). These data are also reported to the European Surveillance of Veterinary Antimicrobial Consumption project (ESVAC), which was launched in 2009 by the European Medicines Agency (EMA), following a request by the European Commission to develop an approach for the harmonized collection and evaluating of antimicrobial usage (AMU) sales data in animals in the member states (8).

In April 2014 the 16th amendment of the German Medicinal Products Act (AMG) was introduced, which requires farmers that keep fattening animals to report their usage of applied veterinary medicinal products with antibiotic components on a half yearly basis (9). To comply with legal requirements, the use of medicines in livestock animals per-production type is recorded by farmers and veterinarians directly in one specific national database (Herkunftssicherungs- und Informationssystem für Tiere). There, data are collected separately for each production type of fattening cattle, pigs, chicken and turkey to determine a farm-specific half-year treatment frequency (TF). Based on these, semiannually the BVL determines the median and third quartile of the TF for each of these livestock populations, which is the basis for further actions, such as consulting the veterinarian or writing an action plan to reduce AMU (9).

Monitoring systems that pursue economic or scientific interests were also introduced in Germany. The private company “QS Qualität und Sicherheit GmbH” (QS) offers a benchmark system on farm level in Germany for poultry, pigs, and calves for fattening (10).

In the frame of the scientific projects VetCAb and VetCAb-Sentinel (VetCAb-S), the antibiotic usage at farm level is determined by used quantities and number of applied single doses. In the latter project not only the usage of antibiotics in livestock used for fattening, but also of dairy cows and dairy calves is recorded and evaluated.

The aim of this work is to present the results of data analysis on antibiotic usage in dairy cows, dairy calves and beef cattle in the years 2011, 2013, 2014, and 2015. Moreover, the association between temporal trends and the factors “farm size,” “region,” and “veterinarian” on the AMU is analyzed.

## Materials and Methods

### Study Population and Data Collection

Data for 2011 were collected within the pilot phase of the VetCAb project with a cross-sectional approach (11). To ensure a cross-sectional study like study population the data was checked for its representativeness by investigating the demographic characteristics of the participating farms by comparing these with official data of the agricultural statistics (12). Since 2013, the project is continued as a longitudinal study with ongoing participant recruitment, called VetCAb-S (13). The study population was initially recruited as a convenience sample by addressing all veterinarians and farmers by general information in newsletters and the German Veterinary Record (“Deutsches Tierärzteblatt”), which is sent out mandatorily to all veterinarians in Germany. Farmers and veterinarians voluntarily provide AMU data via ADF about the number of animals treated, date and duration of treatment, name and amount of the medicinal product used, indication and application route (14). Information on the number of livestock places, i.e., the animal capacity of the individual farms, is requested separately. After checking completeness and pharmacological plausibility as previously described (15), data are included in the evaluation.

In this survey, three production types are considered: dairy cows, dairy calves and beef cattle. Dairy cows are defined as cows kept for milk production. The group of dairy calves includes calves reared on dairy farms for later use as dairy or beef cattle. The number of livestock locations for dairy calves is not collected directly, it is assumed as the number of livestock locations for dairy cows that are kept on the farm. Beef cattle are defined as cattle from 8 month old, reared for meat production. Because each participating farm can keep one or more production types, the allocation to the respective groups is mainly based on the category given on the ADFs.

### Measuring Antibiotic Usage

In order to quantify antibiotic usage, the number of antimicrobial substance applications (number of used daily doses, nUDD) is determined using the records in the database as follows:

$$\begin{array}{l} nUDD=number\;of\;animals\;treated\;\times \;number\;of\;days\;treated\\ \;\times number\;of\;active\;ingredients \end{array}$$

By means of the TF, the average number of treatments per animal of the observed population within a given time period is calculated (16–18):

$$\begin{array}{l} TF=\;\frac{nUDD}{farm\;size} \end{array}$$

Following the general rules of the AMG, the measurements for all applications are calculated for each holding per half-year. Treatment of udder diseases and all treatments in the context of dry-cow therapy are included in the evaluations. Each production type kept on a farm within half a year is defined as a holding in the analysis. In the project, the reference population is defined by number of available livestock places per holding. The population under study is herein referred to as the “study collective.” When entering the study collective, the number of livestock locations of dairy cows and beef cattle of every farm was recorded. This information serves as a basis for calculating the TF over the entire period.

### Statistical Analysis

Two statistical model evaluations were applied. In order to analyse whether there are trends in the development of the TF over time, linear and quadratic trend effects of the TF were calculated with polynomial regression by orthogonal polynomial coefficients within linear models. Due to different sub-trends within the data, the calculations were carried out over two periods, based on the first six and on all eight considered half-years from 2011 to 2015.

In a second approach, the general impacts of the factors “time,” “farm size,” and “region” on antibiotic usage in dairy cows and dairy calves were considered using multiple generalized linear mixed regression models for calculating a three-way ANOVA with nested subjects, using the TF as the outcome. For this purpose, a right-trimmed data set was used to guarantee robust model estimators, where the top 1% TFs were excluded (19). The same method has already been used on pigs (15). As the antibiotic usage is measured semi-annually, there are eight observations per holding within the analyzed time period. The missing year 2012 leads to different intervals between the regarded time points. A flexible correlation structure between observations of one farm is chosen due to the non-equidistant time points. The estimated covariance parameters showed that covariance between time points 2011–1 and 2011–2 with others are smaller than those of later time points. Therefore, a structure with constant covariance or e.g., auto-regressive structures are not suitable. The choice of the variance structure affects the model estimates of variances and consequently the observed confidence intervals and *p*-values. The factor “farm size” was categorized into three groups by means of the 33- and 66%-percentile of the number of livestock places per holding on the basis of study population in 2011. The cut-offs for dairy cows were 59 and 116, for dairy calves were 55 and 114, and for beef cattle 35 and 60 livestock places, respectively. For data analysis referring to the factor “region,” the examined collective of cattle farms is divided into three geographical areas (Middle, Northwest and East Germany) based on agricultural structures in Germany (20). Only a small proportion of participating beef cattle holdings from the eastern region has been documented, therefore these holdings were not considered for evaluation. According to Hemme et al. (15), the impact of the veterinarian on the TF was taken into account as a random effect following a hierarchical model structure (15). Compound-symmetry covariance structure for the modeling of the random veterinary effect was assumed. Impact of the veterinarian random effect was analyzed by using a likelihood ratio chi-square test comparing the full model with the reduced model, thus omitting the hierarchical level. We considered three different regression models for evaluation in terms of transformation due to a skewed distribution of residuals: square root transformation, logarithm transformation after adding 0.1 and logarithm transformation after adding 1. Results were converted to the original scale after retransformation of least-squares means with associated 95% confidence intervals. The residuals of the final models were distributed normally. Due to zero inflated data of beef cattle, an appropriate result regarding the distribution of the residuals could not be achieved when comparing the different transformations. Therefore, no adequate model for the TF could be adapted. Hence, we conducted a multi-factorial mixed logistic regression to model the antibiotic usage. In the logistic regression, the odds-ratio confidence intervals were calculated to describe the effect of risk factors. The estimation was done applying the Residual Pseudo Likelihood method. The 95% confidence intervals for parameters of interest were reported.

The analyses were performed in SAS, version 9.3 TS level 1M2 (SAS Institute Inc., Cary, NC, United States), using the procedures MIXED and GLIMMIX, respectively, and entailing F-tests to assess the statistical significance of fixed effects. *P*-values below 5% were considered as statistically significant.

## Results

### Study Population

During the observational period, a total of 95,944 ADFs from participating dairy and beef cattle farms for the years 2011, 2013, 2014, and 2015 were evaluated. Of these, 79,528 ADFs were allotted to dairy cows, 14,424 ADFs to dairy calves and 1,992 ADFs to beef cattle. Due to the two project phases, pilot and sentinel study, a drop in the number of participating dairy cow and dairy calf holdings was evident between 2011 and 2013. Seventeen percent of the dairy cow and calf holdings and 16% of the beef cattle holdings participated throughout the entire period considered. The other part consisted of holdings, which participated in sections, joined the collective later than 2011 or left the collective earlier than 2015. The discrepancy between the analyzed number of cow and dairy calf holdings resulted from the trimmed 1% of the semi-annual TF and disregarded holdings, respectively. At the beginning of the sentinel study in 2013, the number of participating beef cattle holdings could be increased and then kept at a constant level (see Table 1). Within the study collective, the following numbers of antibiotic substance prescriptions were made per holding half-yearly in the median: 27 for dairy cows (Interquartile range (IQR) = 12–48 prescriptions per holding), three for dairy calves (IQR = 1–8 prescriptions per holding) and one for beef cattle (IQR = 0–3 prescriptions per holding). Most of these holdings were located in northwest Germany, followed by holdings from the middle and east of Germany.

**Table 1:** Distribution of the treatment frequency per half-year for dairy cows, dairy calves and beef cattle.

**Half-year**	**Number of holdings**	**Semi-annual treatment frequency**						
		**Minimum**	**5%-quantile**	**25%-quantile**	**Median**	**75%-quantile**	**95%-quantile**	**Maximum**
**DAIRY COWS**								
2011-1	474	–	0.3	1.1	1.9	3.2	5.7	11.7
2011-2	474	–	0.3	1.2	2.2	3.4	6.0	11.5
2013-1	178	–	0.0	1.1	2.1	3.5	6.3	11.3
2013-2	175	–	0.1	1.2	2.1	3.4	6.0	10.8
2014-1	173	–	–	1.2	2.1	3.3	7.5	12.7
2014-2	170	–	0.3	1.3	2.3	3.6	6.8	12.7
2015-1	177	–	–	1.0	1.9	3.1	6.5	12.1
2015-2	178	–	–	1.1	2.2	3.8	7.7	12.7
**DAIRY CALVES**								
2011-1	473	–	–	0.1	0.4	2.1	7.6	20.2
2011-2	473	–	–	0.1	0.5	2.5	9.0	20.8
2013-1	177	–	–	0.1	0.6	2.1	9.5	16.7
2013-2	175	–	–	0.0	0.6	3.1	10.1	20.0
2014-1	173	–	–	0.1	0.6	2.7	8.6	22.6
2014-2	171	–	–	0.1	0.8	3.0	11.3	22.9
2015-1	179	–	–	0.0	0.6	1.6	7.6	13.1
2015-2	179	–	–	–	0.3	1.6	6.3	16.4
**BEEF CATTLE**								
2011-1	45	–	–	0.0	0.2	0.6	1.8	2.7
2011-2	45	–	–	–	0.1	0.2	0.7	5.3
2013-1	76	–	–	–	0.1	0.5	8.7	16.0
2013-2	76	–	–	–	0.1	0.3	6.5	22.4
2014-1	75	–	–	–	–	0.6	13.1	34.7
2014-2	75	–	–	–	–	0.3	6.4	20.5
2015-1	79	–	–	–	–	0.3	4.3	33.0
2015-2	77	–	–	–	–	0.2	1.8	26.6

–*, observed zero; 0, zero by rounding*.

### Antibiotic Usage and Treatment Frequency

**Figure 1:**
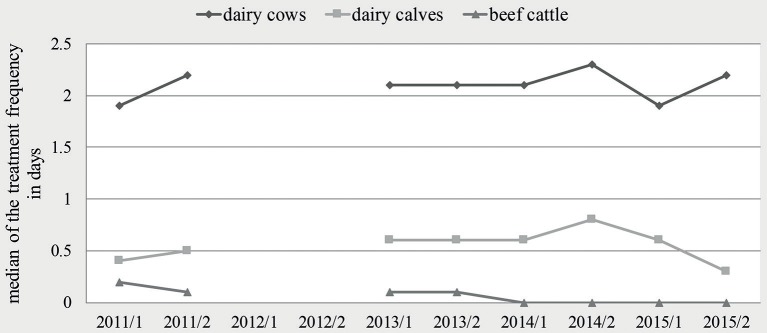
Median of the treatment frequency per half-year for dairy cows, dairy calves and beef cattle.

Table 1 shows the distribution of the semi-annual TF of dairy cows, dairy calves and beef cattle holdings within the observed time period. In dairy cow holdings, the median of the semi-annual TF was quite constant with minor deviations. In dairy calves, the median of the semi-annual TF increased from 0.4 in the first half-year of 2011 to 0.8 in the second half-year of 2014, before dropping to 0.3 in the second half-year of 2015. For beef cattle, a continuous reduction of the median was seen from 0.2 in 2011–1 over 0.1 between the second half-year of 2011 and 2013 to zero from the first half-year of 2014 until the end of the observation (see Figure 1). The proportion of holdings without antibiotic usage increased during the whole observed time period in all three production types. The most obvious change occurred in beef cattle holdings; here, the proportion of holdings without antibiotic usage increased from 22.2% in the first half-year of 2011 to more than half of the participating beef cattle holdings (54.5%) in 2015–2. In dairy cows, the proportion increased from 1.3 to 11.2%, and in dairy calves from 16.1 to 25.1%. Regarding the production types dairy cow and dairy calf, a trend test over the first six half-years showed an increasing linear trend (dairy cows: *p* = 0.017; calves: *p* = 0.004). If the time-period was extended to all eight half-years under study, this turned into a quadratic effect (dairy cows: *p* = 0.006; calves: *p* < 0.001). For beef cattle, this model approach was not feasible due to a large extend of zero antibiotic usage in this production type. Therefore, zero inflated data was observed and no computable results were reported here.

### Regression Models

For dairy cows and dairy calves, linear regression models with different transformations were applied to assess the impact of several factors on the farm specific semi-annual TF. The best results for dairy cows were achieved using the square root transformation. Table 2 shows the effects of each variable, the mean TF in the corresponding category, as well as the associated 95% confidence intervals. The calculations show that only the general factor “time” had a statistically significant impact on the TF in dairy cows. The random factor “veterinarian” had a statistically significant impact on the TF in dairy cows (*p* < 0.001).

**Table 2:** Results of the multi-factorial model with square root transformation for the treatment frequency in dairy cows.

**Factor**	**Category**	**N**	**Mean**	**CI_l**	**CI_u**	***F*-value**	***p*-value**
Half-year	Global					4.348	<0.001
	2011-1	474	1.783	1.371	2.249		
	2011-2	474	1.962	1.529	2.448		
	2013-1	178	2.109	1.629	2.649		
	2013-2	175	2.079	1.603	2.618		
	2014-1	173	2.006	1.534	2.542		
	2014-2	170	2.231	1.728	2.797		
	2015-1	177	1.783	1.327	2.307		
	2015-2	178	1.954	1.451	2.532		
Farm size	Global					1.174	0.324
	Lower third	607	1.856	1.380	2.403		
	Middle third	647	2.076	1.589	2.630		
	Upper third	745	2.028	1.557	2.561		
Region	Global					2.087	0.195
	Middle	490	1.750	1.232	2.358		
	Northwest	1343	2.131	1.541	2.815		
	East	166	2.088	1.236	3.161		

*CI_l, CI_u, Lower and upper limit of the 95% confidence interval*.

For dairy calves, the best fit of the model was observed using the logarithm transformation after adding 0.1. In addition to the factor “time,” the factor “farm size” had a statistically significant effect on TF in dairy calves (see Table 3), and the average estimator increased with increasing farm size. Between farms in the middle and lower thirds of farm size, an increase of the mean estimator from 0.46 to 0.70 and a clear shift of the confidence interval was evident. The factor “veterinarian” had a statistically significant impact on TF in dairy calves (*p* < 0.001). The distributions of the residuals of multi-factorial models with different transformations for the TF for dairy cows and dairy calves are available as Supplementary Data.

**Table 3:** Results of the multi-factorial model with logarithm transformation for the treatment frequency in dairy calves.

**Factor**	**Category**	**N**	**Mean**	**CI_l**	**CI_u**	***F*-value**	***p*-value**
Half-year	Global					3.606	0.003
	2011-1	473	0.442	0.295	0.643		
	2011-2	473	0.500	0.338	0.723		
	2013-1	177	0.550	0.359	0.820		
	2013-2	175	0.601	0.389	0.907		
	2014-1	173	0.648	0.427	0.963		
	2014-2	171	0.661	0.433	0.988		
	2015-1	179	0.443	0.282	0.671		
	2015-2	179	0.385	0.241	0.589		
Farm size	Global					6.375	0.005
	Lower third	672	0.433	0.272	0.665		
	Middle third	592	0.462	0.295	0.698		
	Upper third	736	0.701	0.472	1.022		
Region	Global					1.167	0.365
	Middle	484	0.423	0.248	0.686		
	Northwest	1343	0.547	0.323	0.890		
	East	173	0.609	0.264	1.281		

*CI_l, CI_u, Lower and upper limit of the 95% confidence interval*.

In beef cattle, a logistic regression model was adapted. Table 4 shows the results for beef cattle farms with antibiotic use in general (yes vs. no) and estimated odds ratios with associated 95% confidence intervals. Results show that “time” and “farm size” had a statistically significant impact on the AMU in general. The odds ratios decreased until second half of 2014, which suggested a reduction of the odds to use antibiotics in comparison to odds of not using antibiotics. The odds to use antibiotics in farms of the upper third was 2.8-fold higher than in farms of the lower third. No statistically significant impact of the factor “veterinarian” on the TF in beef cattle holdings could be determined (*p* = 0.674).

**Table 4:** Results of the multi-factorial logistic regression model for the treatment frequency in beef cattle.

**Factor**	**Category**	**N**	**Use %**	**Odds ratio**	**CI_l**	**CI_u**	***F*-value**	***p*-value**
Half-year	Global						6.251	<0.001
	2011-1 (ref.)	45	77.78	1.000				
	2011-2	45	71.11	1.634	0.579	4.609		
	2013-1	77	56.58	0.522	0.253	1.076		
	2013-2	76	52.63	0.446	0.260	0.764		
	2014-1	75	45.33	0.288	0.137	0.607		
	2014-2	75	37.33	0.217	0.109	0.433		
	2015-1	79	49.37	0.402	0.231	0.701		
	2015-2	77	45.46	0.319	0.164	0.621		
Farm size	Global						9.987	<0.001
	Lower third (ref.)	268	38.43	1.000				
	Middle third	112	57.14	0.898	0.513	1.571		
	Upper third	169	70.83	2.814	1.653	4.793		
Region	Global						5.377	0.073
	Northwest (ref.)	366	58.90	1.000				
	Middle	183	38.80	0.461	0.191	1.114		

*CI_l, CI_u, Lower and upper limit of the 95% confidence interval*.

The factor “region” had no statistically significant impact on the semi-annual TF in none of the three production types. The estimates of fixed effects regression coefficients and random effects covariance parameters are provided in the Supplementary Material.

## Discussion

Within the longitudinal study VetCAb data from dairy cows, dairy calves, and beef cattle were observed over several years, facilitating an examination of temporal trends in AMU. For this purpose, a calculated semi-annual TF for each holding was used, based on data at farm level. The impact of factors like farm size and region on antibiotic usage was investigated via regression models.

The output of the study presented here is based on voluntary participation, which carries the risk of a selection bias. The number of farms enrolled were proportional to the German farm demographics and therefore a larger number of dairy and a lower number of beef cattle farms were included (12). Due to ongoing participant recruitment, there were changes in the population of study participants, which is typical for open cohort studies. In relation to the number of participating dairy holdings, there was a drop from the pilot to the sentinel study. This decline in participants could not be compensated by new recruitments, and this has to be taken into account when interpreting the smaller collective from 2013 on. In contrast, the number of participating beef cattle farms increased during the observational period. This may be due to the fact that in 2014 the legal monitoring of AMU in fattening animals was introduced in Germany (9). In beef cattle farms, the majority of antimicrobial use data are transmitted online from the software of veterinary practices to the governmental monitoring system. Hence beef cattle holdings could use the same data set to participate in the study with little additional efforts.

As data from routine documentation are used, this carries the (“practical”) risk of misallocations to the incorrect production type group. Especially for calf rearing production type, designations were not standardized. Generally, distinctions should be made between calves reared for dairy heifer replacements, calves reared for beef production, and calves fattened for veal production (21, 22). The analyzed dataset contains calves reared on dairy farms for later use as dairy cows or beef cattle. This production type has to be differentiated between calves, which are reared and slaughtered for veal production. When interpreting the results, it should be noted that within the group of dairy calves there is an inhomogeneity of later more clearly separable production type groups. However, the risk of misclassification was minimized by taking into account the type of the farm reflected in the production types included in the database, accompanied by regular communication with the farmers. Concerning dairy cows and beef cattle, misallocations to the production type groups were unlikely.

To measure the usage of antimicrobial agents, which was calculated on the basis of the number of used daily doses (nUDD). This type of calculation was possible because the information needed is maintained in the ADFs by official regulation in Germany. As described before, ADFs provide detailed information on the actual number of animals treated, number of treatment days and the total amount of antimicrobials used (11). To draw conclusions about the correctness of dosages by comparing the UDD with the labeled dose, additional information is necessary e.g., details of the indication and the veterinarian's decision process, which were not included in our data.

As stated by Pinto Ferreira et al. (23), collecting real use data at farm level is at this time the most accurate way to monitor AMU, because only recording the actual use contributes to avoidance of approximations and resulting data distortion (23). Monitoring systems for AMU at farm or prescriber level provide the opportunity to guide individual preventive or corrective management actions (24). The calculation here is in line with the general therapy incidence concept (25), but real nUDD is used instead of nDDD (number of defined daily doses) and implicit body weight under treatment is used instead of standardized body weights (17). Half-yearly information on the number of livestock places of a holding was not available throughout the project. Therefore, the number of livestock places initially recorded was taken as a basis for calculation (15). We anticipate that the resulting bias is negligible, as we know from transnational data, that the average number of cattle per farm has barely changed over the years considered in Germany (26). Between 2013 and 2015, the half-yearly average number of cattle per farm was 80, 80, 82, 82, 84, and 84, respectively (26). The number of livestock locations for dairy calves is assumed as the number of dairy cows per farm and year. However, it should be taken into account that the period each calf spends on a dairy farm differs from farm to farm. Assuming that this inaccuracy is not related to the number of treatments, it would lead, if at all, to a non-differential information bias. We believe that this assumption is justified, considering the conditions in calf rearing in Germany.

Given the differences of national monitoring systems, transnational comparisons are primarily made based on sales data. In the framework of the ESVAC project a 53% decrease in the overall sales of veterinary antimicrobial agents in Germany between 2011 and 2015 was reported (7). Trends in sales data from other European countries, e.g., Denmark, Belgium, and the Netherlands, showed an obvious reduction of AMU, as well (27). Within the ESVAC project, there is a cross-species documentation of the quantities sold, and it is not possible to allocate the amounts of sold quantities to individual animal species, animal age categories or production types (23, 28). Because an exclusive interpretation of quantities sold cannot provide detailed information on the use of antibiotics, projects and studies of several countries are trying to quantify consumption more closely.

### AMU in Dairy Cows

Since dairy cows are not included in the official German antibiotic monitoring system (9), ADFs of dairy farms are collected and analyzed only within this study in Germany. Reporting AMU in dairy cows in the QS-system is based on a small voluntary part of the members only. Therefore, no results have been reported so far (29). Our results show the determined half-year TF ranges between 1.9 and 2.2 days with minor deviations. Compared with the TF calculated for different production types in pigs within the VetCAb-study by Hemme et al. (15) for the same time period, the use of antimicrobials in dairy cows appears to be low but varies over time (15). Merle et al. (30) identified a TF of 0.85 days per 100 days within the VetCAb feasibility study for dairy cows (30). Regarding the shorter observation period, this result corresponds to our results; no temporal trends were identified within this study.

Denmark reports the overall consumption in cattle remained constant between 2011 and 2015 (31). It is emphasized that the vast majority of cattle biomass is comprised by dairy cows, which have a low consumption of antimicrobial agents compared to growing animals (31). In addition to the analysis of sales data, the amount of antibiotics is documented via prescription records including information on animal species, age-group and diagnostic grouping (VetStat). In the annual report, the antimicrobial agents sold for cows and bulls is put together, but that comparability is not given here. To reduce treatment of clinical mastitis the Danish Cattle Association introduced the “milk quality campaign” in 2010 (31).

Using prescription records as data source for AMU is standard practice in the Netherlands as well. The Netherlands Veterinary Medicines Institute (SDa) reports AMU in the Netherlands in dairy farms separately from other cattle. Data is presented as overall antibiotic use, use of dry cow antibiotics, use as mastitis injectors as the defined daily dose animal at farm level (DDDA_F_). In 2012, 2013, 2014, 2015 the annual median DDDA_F_ was 2.7, 2.8, 2.2, and 2.1, respectively (32). This seems to be on a similar level, although the TF is working with UDDs and therefore these measures were not comparable directly.

Belgium has also achieved a reduction in antibiotics used in the veterinary field in general between 2011 and 2015 (33). In our study as well as in other studies (30, 34–36), it appears that bovine mastitis is by far the most common indication in dairy cows and reason for treatment with antimicrobial agents (37). In line with this, within the considered period, the majority of antibiotic prescriptions in cattle were dedicated to dairy cows. A Swedish study reported that the treatment of dairy cows constitutes the largest proportion of antibiotic drugs in dairy production, as well (38). The present evaluations include treatment of udder diseases and all treatments in the context of dry-cow therapy. An Austrian study evaluated AMU data with respect to udder diseases of 248 dairy farms in Austria within a 1 year period in 2015 and 2016. The determined mean number of Defined Daily Doses for animals (DDD_vet_) per cow and year was 1.33 (34). In this study population, treatments for udder disease made up 36.4% of all antimicrobial treatments. Considering that within these evaluations dry cow therapy was excluded, these results are largely consistent with our results. Since it is well-known that antimicrobial substances applied intramammary for dry cow therapy make up a large proportion of the antibiotic consumption in milking cows (39), research with respect on these different treatment options is needed.

### AMU in Dairy Calves

Reporting of AMU in dairy calves not reared for veal or beef production is not mandatory in Germany. Therefore, no direct comparisons to the compulsory system are possible. Our study results show that the median of the TF of dairy calves increases continuously from 0.4 to 0.8 until the 2nd half of the year 2014 and decreases to 0.3 within the year 2015. Antimicrobial agents sold (kg active compound) in Denmark for calves increased between 2012 and 2015. However, except for the age (<12 month), the group of calves is not further determined (31). The reported median of antibiotic use in DDDA_F_ reported by the Dutch Veterinary Medicine Authority in calf rearing farms in the Netherlands since 2013 is zero (40). Disparities with our results in that case can be explained by different national definitions of the production groups on the basis of gender and age. Due to differing definitions within this production type group, direct comparisons in relation to AMU are not feasible. Consistent with our results, a Swedish study mentioned before that, compared to the treatment of dairy cows, overall drug use for dairy calves is at a low level (38) and used to treat mainly respiratory and digestive diseases if necessary, antimicrobials may be administered in calves orally or by injection (35, 37). Though factors like transport and stress contribute to an increased risk of infectious diseases and become an important determinant of antimicrobial use (41).

### AMU in Beef Cattle

In our study over the course of time, the majority of participating farms reduced their use of antibiotics calculated as TF to zero. Compared to the TF of dairy cows, the median of the semi-annual TF was at a very low level already at the beginning of the study and decreased further from 2014 onwards. At the end of the period considered, more than a half (54.5%) of the participating farms did not use any antibiotics at all. Our results are in line with the nationwide monitoring of antibiotic use in beef cattle: the median and the third quartile of TF are zero (42–44). The QS-system for beef cattle shows similar results, too (QS). Therefore, it can be assumed that in the present collective a serious selection bias is unlikely. The reported median of antibiotic use in DDDA_F_ reported by the Netherlands Veterinary Medicines Institute in beef farms is zero since 2013, as well (40).

### Factors Associated With AMU

Several studies have already examined associations between factors such as farm size, region, disease incidence and antibiotic usage in cattle (45–48). To put these factors in relation with the AMU data of cattle within the VetCAb collective, regression models have been calculated for each production type. Hence, mapping the effects of farm size, region and the veterinarian in a temporal context is facilitated.

In dairy cows, the estimated means of the TF rose with the increasing farm size in this study. However, the results of the model also demonstrate that there is no statistically significant impact of farm size on TF. Gonzaley Pereyra et al. (45) observed no significant association between herd size and antimicrobial use in dairy cows from 18 milking herds, as well (45). In contrast, an increase in subclinical mastitis with increasing numbers of cows on Swiss dairy farms was found by Doherr et al. (49). Hill et al. studied dependencies between herd size and antimicrobial treatments of diseases like mastitis and lameness on dairy operations in the United States and found that with increasing herd size, herd-level disease prevalence increased. However, with increasing herd size within-herd prevalence seemed to decrease (46).

In calves, the estimated means of the TF rose with increasing farm size in this study, showing a statistically significant impact of farm size on the TF. These results are in line with the results of other studies: the purchase of calves from dairy farms is common and known to be one of the biggest risk factors for disease in dairy calves (50). Most of the indications for antibiotic treatment in calf production are linked with respiratory disease and enteritis (37). Frequency of respiratory tract infections have also been linked with larger calf group sizes (51). Here a direct comparison is not given, since the group size in which the calves are held was investigated and not the total number of livestock locations. Summarizing this, our findings on the impact of farm size on the frequency of antibiotic treatments seem plausible due to usual management practices in calf rearing.

For beef cattle, a very small number of antibiotic treatments were documented within the considered time period. Consequently, a logistic regression model was calculated, to estimate the overall chance of AMU in relation to a given reference. However, the number of animals treated, the duration of treatment and the frequency of application are not included in the model. Results show a statistically significant impact of farm size on the AMU in beef cattle. Beef cattle are kept in groups and the purchase of calves from several stocks is common (41). It can be assumed that consequently in larger beef herds the possibility for pathogenic exchange and the risk of infectious diseases increases. To the best of the authors' knowledge, research on the impact of farm size on antimicrobial treatments in beef cattle is limited and further studies are needed.

Taking into account structural differences in terms of livestock density and forms of animal husbandry, a regionalization of Germany into agriculturally structurally typically regions was carried out (20). Although it is assumed that a region may be a surrogate for management-related differences due to environment, geography, weather and resources availability that might affect AMU (52, 53), our study showed that in all three analyzed production groups the factor region has no statistically significant impact on the TF.

Pursuant to the current model calculations, the veterinarian has a statistically significant impact on the TF in dairy cows and dairy calves. Possible reasons could be different specializations and experiences of the veterinarians, related to individual prescription behavior influenced by multiple factors like different treatment durations and selection of drugs (54). Regarding prescribing behavior, Speksnijder et al. found that an increasing experience of the veterinarian is associated with being less concerned about possible veterinary contributions to AMR and also being less concerned to prescribe antibiotics to prevent animal diseases (55). In another study, Gibbons et al. determined by means of a questionnaire the factors influencing the choice of the antimicrobial prescribed. It emerged that the majority of surveyed veterinarians (95.7%) considered that the choice of antimicrobial prescribed “often” or “always” was influenced by the veterinary's prior experience of using a drug for a specific condition (56). However, Cattaneo et al. found a negative relationship between years of practical experience and knowledge about consequences of AMR in bovine veterinarians (57). Our results of the logistic regression model show that the veterinarian has no statistically significant impact on the general AMU in beef cattle. This can be explained by the fundamentally different approaches of the two regression models: in context of the treatment of infectious diseases pursuant “good veterinary practice” (58) and corresponding guidelines (59, 60), veterinarians may differ supposedly more in terms of dosage and duration of antimicrobial treatment, as in terms of whether an antimicrobial drug should be applied. Nevertheless, it should be noted that this factor is not adequately investigated in this study and was only modeled as a variable effect within the regression models.

In general, results demonstrate large differences in antimicrobial usage patterns between the production types in bovine livestock. The fact that the compared studies come up with different findings is likely due to the attributes of the particular study population in terms of age groups and production sectors used in each study. Production type specific antibiotic usage data is providing the basis for risk assessment and the recommendation of appropriate countermeasures for prevention of AMR. The results of the present survey emphasize the need for monitoring and evaluating each cattle production sector separately, considering the respective characteristics (61).

## Conclusions

According to our study results, antimicrobial use in dairy cow and dairy calf holdings in Germany varied on a low level across the period observed. In beef cattle holdings a reduction in antimicrobial usage was evaluated. To enable comparisons of the magnitude of antibiotic consumption across regions or countries, production groups should be defined more clearly. Furthermore, as “farm size” has a statistically significant impact on the magnitude of consumption of antibiotics, this should be regularly considered over time. To achieve the overall objective, the reduction of antimicrobial usage and antimicrobial resistance, science based actions need to be taken, reviewed, and adjusted if necessary taking into account the accompanying variables. Regular adaptation of monitoring and benchmark systems is a crucial element in this effort.

## Data Availability

The data were collected on an individual basis from farmers and veterinary practitioners. Each participant gave written consent with the understanding that data would not be transferred to a third party. Therefore, any data transfer to interested persons is not allowed without an additional formal contract. Data are available to qualified researchers who sign a contract with the University of Veterinary Medicine Hannover. This contract will include guarantees to the obligation to maintain data confidentiality in accordance with the provisions of the German data protection law. Currently, there exists no data access committee or another body who could be contacted for the data. But for this purpose, a committee will be founded. This future committee will consist of the authors as well as members of the University of Veterinary Medicine Hannover and members of the funding institution (Federal Institute for Risk Assessment). Interested cooperative partners, who are able to sign a contract as described above, may contact: LK, lothar.kreienbrock@tiho-hannover.de.

## Ethics Statement

Data used within this study is based on mandatory application and delivery forms (ADF), which was provided voluntarily by farmers and veterinarians, signing individual written consent data to be used by the study team only. Our research does not involve any regulated animals and there were no scientific procedures performed on animals of any kind. For this reasons a formal approval by an ethical committee was not necessary under the provisions of the German regulations.

## Author Contributions

KH and LK: conceptualization, formal analysis, investigation, and writing—original draft. KH and MH: data curation and validation. LK: funding acquisition and supervision. KH, IR, MH, and LK: methodology. KH: project administration. MH: software. KH, NW, AK, and LK: writing—review and editing.

### Conflict of Interest Statement

The authors declare that the research was conducted in the absence of any commercial or financial relationships that could be construed as a potential conflict of interest.

## Supplementary Material

Image S1 Distribution of the residuals of the multi-factorial model with logarithm transformation after adding 0.1 for the treatment frequency in dairy cows.

Image S2 Distribution of the residuals of the multi-factorial model with logarithm transformation after adding 1 for the treatment frequency in dairy cows.

Image S3 Distribution of the residuals of the multi-factorial model with square root transformation for the treatment frequency in dairy cows.

Image S4 Distribution of the residuals of the multi-factorial model with logarithm transformation after adding 0.1 for the treatment frequency in dairy calves.

Image S5 Distribution of the residuals of the multi-factorial model with logarithm transformation after adding 1 for the treatment frequency in dairy calves.

Image S6 Distribution of the residuals of the multi-factorial model with square root transformation for the treatment frequency in dairy calves.

## Abbreviations

ADF: Application and Delivery Form
AMG: Medicinal Products Act (“Arzneimittel-Gesetz”)
AMR: Antimicrobial Resistance
AMU: Antimicrobial Usage
BVL: Federal Office of Consumer Protection and Food Safety (“Bundesamt für Verbraucherschutz und Lebensmittelsicherheit”)
ESVAC: European Surveillance of Veterinary Antimicrobial, Consumption
IQR: Interquartile Range
(n)DDD: (number of) Defined Daily Dose(s)
(n)DDDA_F_: (number of) Defined Daily Dose(s) Animal at farm level
(n)UDD: (number of) Used Daily Dose(s)
TF: Treatment Frequency
QS: Qualität und Sicherheit GmbH
VetCAb-S: Veterinary Consumption of Antibiotics—Sentinel.
