# Supplementary figures and images for: Monitoring Antibiotic Usage in German Dairy and Beef Cattle Farms—A Longitudinal Analysis
Source: Front Vet Sci. 2019 Jul 26;6:244. doi: 10.3389/fvets.2019.00244 (PMC6676220; doi:10.3389/fvets.2019.00244)

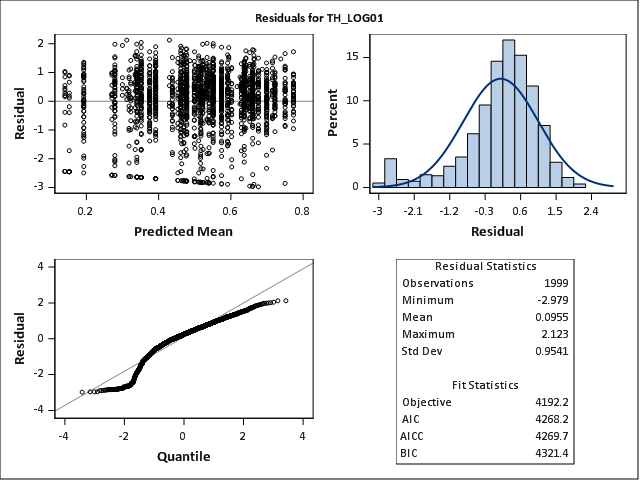

Supplement: Image S1 — Distribution of the residuals of the multi-factorial model with logarithm transformation after adding 0.1 for the treatment frequency in dairy cows. [file Image_1.PNG]

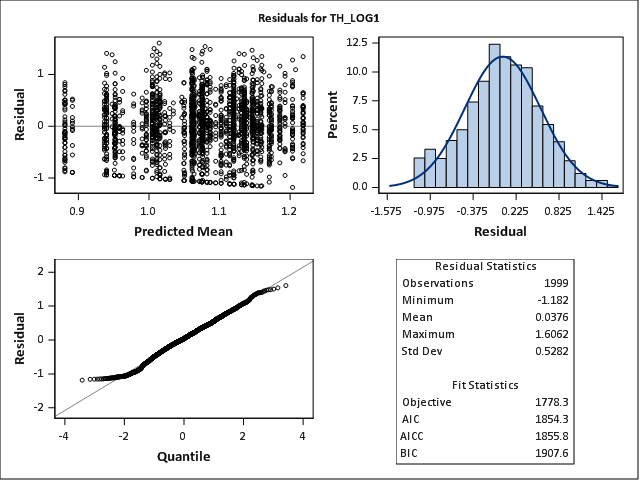

Supplement: Image S2 — Distribution of the residuals of the multi-factorial model with logarithm transformation after adding 1 for the treatment frequency in dairy cows. [file Image_2.PNG]

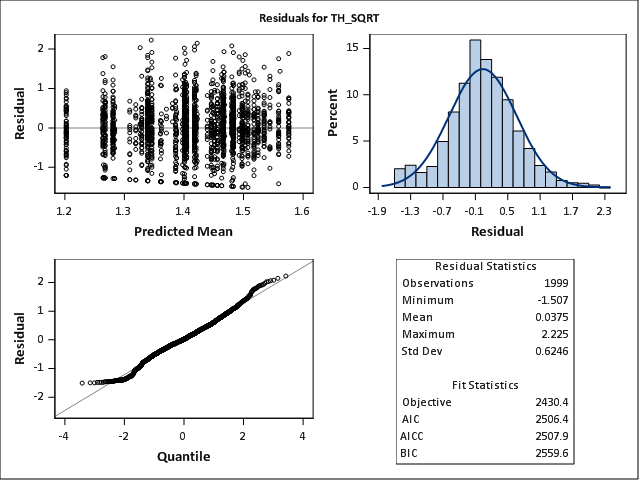

Supplement: Image S3 — Distribution of the residuals of the multi-factorial model with square root transformation for the treatment frequency in dairy cows. [file Image_3.PNG]

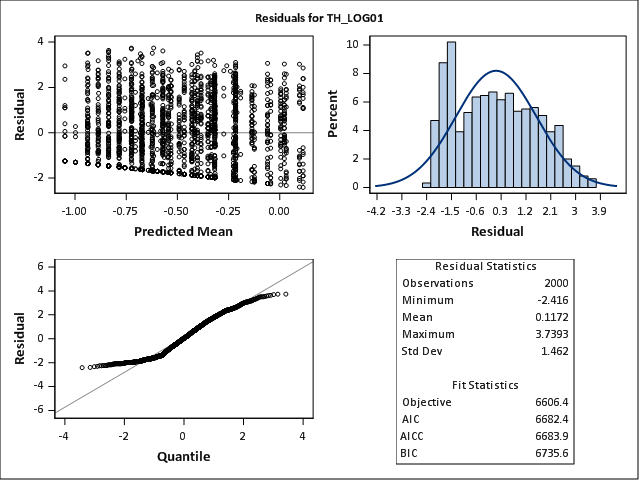

Supplement: Image S4 — Distribution of the residuals of the multi-factorial model with logarithm transformation after adding 0.1 for the treatment frequency in dairy calves. [file Image_4.PNG]

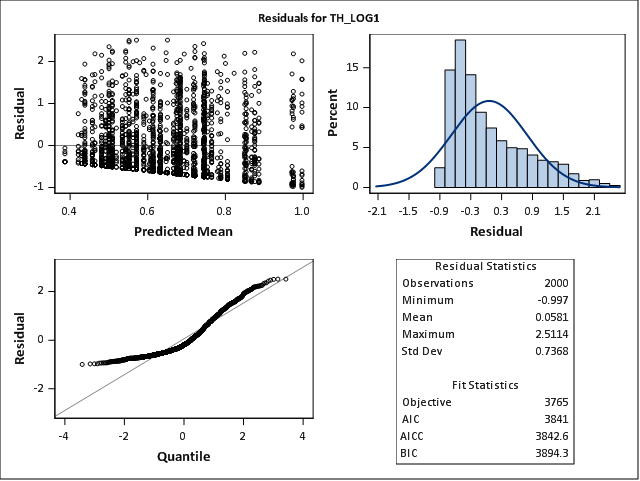

Supplement: Image S5 — Distribution of the residuals of the multi-factorial model with logarithm transformation after adding 1 for the treatment frequency in dairy calves. [file Image_5.PNG]

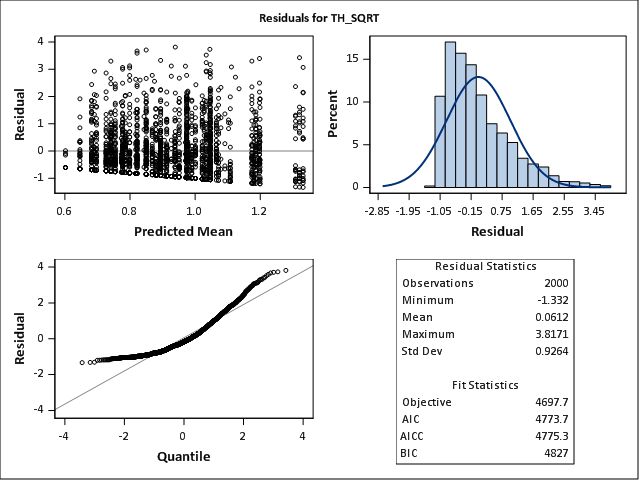

Supplement: Image S6 — Distribution of the residuals of the multi-factorial model with square root transformation for the treatment frequency in dairy calves. [file Image_6.PNG]
